# Comparison of interfragmentary compression across simulated condylar fractures repaired using four techniques

**DOI:** 10.3389/fvets.2023.1233921

**Published:** 2023-09-20

**Authors:** Ashley Brabon, Kristopher James Hughes, Raphael Labens

**Affiliations:** Faculty of Science, School of Agricultural, Environmental and Veterinary Sciences, Charles Sturt University, Wagga Wagga, NSW, Australia

**Keywords:** configuration, condylar, equine, fracture, repair

## Abstract

**Introduction:**

Equine condylar fractures are commonly repaired using cortex screws applied in lag fashion. Inadequate interfragmentary compression can lead to post-operative complications.

**Methods:**

Lateral condylar fractures were simulated in 21 cadaver limbs (8 third metatarsals, 13 third metacarpals). In each limb, pressure-sensitive film (Prescale®, Fuji Photo Film Co.) was placed in each osteotomy prior to repair with 4.5 mm diameter cortex screws placed in lag fashion. Screws were placed in linear (L), triangular (T), linear plus a washer (LW) and sequentially tightened triangular configurations (TD1). All screws were tightened to a torque of 4 Nm. Pressure prints obtained were scanned using dedicated software (Fuji FPD-8010E, Fuji Photo Film Co.). A Bayesian Network (BN) model was developed to investigate the impact and interrelationship of each factor on interfragmentary compression. Sixty-three repairs (20*L, 24* T, 11*TD1, and 8*LW) performed on 21 limbs were included in the analysis.

**Results:**

The BN predicted mean contact area (±s.d.) for pressures within the operating range of the prescale film [≥2.5 Megapascals (MPa) ≤ 10 MPa] by L, T, TD1 and LW repairs were 403mm^2^ ± (140), 411 mm^2^ ± (120), 403 mm^2^ ± (120), and 366mm^2^ ± (70). The mean contact area (± s.d.) created by L, T, TD1 and LW repairs at pressures >10 MPa were 112 mm^2^ ± (48), 167 mm^2^ ± (67), 142 mm^2^ ± (50), and 100mm^2^ ± (27). When pressures ≥2.5 MPA to ≤10 MPa were considered, the construct (T or L), washer and screw tightening sequence variables had a very low effect on interfragmentary contact area. At pressures >10 MPa BN sensitivity findings were 16.3, 5.03, and 0.133% for construct, washer and screw tightening sequence. The BN model indicated that triangular repair configuration had a weak influence in the ≥2.5 MPa ≤ 10 MPa range and a moderate influence in the <10 MPa range, on interfragmentary compression. The addition of a washer and the screw tightening sequence had a weak influence on interfragmentary compression at all pressure ranges.

**Discussion:**

The results show that triangular repairs create larger interfragmentary contact areas at greater interfragmentary pressure in simulated condylar fractures, however it is unknown if this results in improved repair stability in the clinical scenario.

## 1. Introduction

Fractures originating from the distal condyles of the third metacarpal or metatarsal bone (condylar fractures) represent the most frequent long bone fracture in horses and are a common cause of wastage in racehorses, world-wide (1, 2). Internal fixation is the recommended treatment for all but the simplest of condylar fractures (3). Among various fixation techniques, fixation with cortex screws placed in lag fashion is most commonly employed for the treatment of condylar fractures (2). Achieving absolute stability is crucial for primary bone healing in the absence of callus formation, which is essential for optimum healing of articular fractures (2, 4). Lag screw fixation achieves fracture stabilization through compression alone (5). Absolute stability is attained when the interfragmentary friction produced is greater than the traction exerted by the limb’s function (4). A screw applied in lag fashion engages the remote cortex only, and the approximation of the screw threads within the remote cortex, and the screw head, results in interfragmentary compression (4). Various methods have been used to assess interfragmentary compression in a variety of osteotomy models, including load cells, pressure sensitive washers, pressure sensitive film and strain gauges (6–9). Despite previous investigations, the optimum interfragmentary compression for repair of condylar fractures is unknown. Although, pressure necrosis, as a result of excessive interfragmentary compression, has not been demonstrated in sheep osteotomy models, and is considered unlikely to occur in horses, it should be considered (2, 5). Common technical errors encountered during the repair of condylar fractures include, inadequate compression of the fracture and imperfect anatomical fracture reduction (10). Inadequate interfragmentary compression along the articular margin and imperfect anatomical reduction can predispose the joint to excessive postoperative osteoarthritis and poor outcomes (10, 11). Increasing the amount of torque applied to a screw head increases the amount of compression achieved across the fracture plane (12). But, inadequate interfragmentary compression cannot be overcome by increased screw torque alone. Because, lag screw fixation has a low tolerance to single overload, and as a screw is tightened the risk of implant or substrate failure increases (13).

Most commonly, condylar fractures are repaired using a single column of lag screws, placed in accordance with principles developed by the AO foundation (2). However, it has been suggested that using two parallel screws through the distal condyle, can improve the strength of repair (10). Recently, an *ex vivo* study showed that two parallel screws placed in the proximal phalanx, adjacent to the metacarpophalangeal joint, provided greater stability under loaded conditions when compared to a single screw (14). Additionally, triangular repair has been demonstrated as a safe and effective treatment option for managing sagittal fractures of the proximal phalanx in racehorses (15). When multiple screws are employed in a lag repair, it has been suggested that an alternate screw tightening technique should be employed to achieve better interfragmentary compression and limit displacement during internal fixation (16).

A washer can be used to distribute the force applied by the screw head to a larger area of cortical bone, subsequently increasing the torque applied before the screw head breaks through the cortical bone (12). The compression applied by a screw affects a small portion of the surrounding bone, and interfragmentary compression reduces as the distance from the screw increases (5). The addition of a washer to internal fixation constructs has been reported to result in increased interfragmentary compression (12).

The primary goals of surgical repair of condylar fractures include re-establishing articular congruency, reduction of the fracture gap and achieving stabilization (2). Increased interfragmentary compression would improve fracture stability and may reduce postoperative arthritis, in turn contributing to improved results for horses undergoing lateral condylar fracture repair. A cadaveric study was conducted to compare the interfragmentary compression achieved by four different repair configurations. The hypothesis was that interfragmentary compression would be greater for triangular (T) vs. linear (L), linear + washer (LW) vs. L and T vs. sequentially tightened triangular (TD1) configurations, respectively.

## 2. Materials and methods

### 2.1. Limbs

The study used limbs from mature horses, which were subjected to euthanasia for reasons unrelated to the study or to disease in the metacarpophalangeal region. Horses were of mixed sex and breed, but the horse population which makes up the case load of the clinic being predominantly Thoroughbred and Standardbred racehorses (approximately 35% respectively) with the remainder made up of performance and pleasure horses of a variety of breeds. Thirty-four metacarpi and metatarsi were collected, and all soft tissues were removed before the limbs were wrapped in wet towels and stored at −20°C until use. The collection of cadaveric specimens was approved by the animal care and ethics committee (A21378) at Charles Sturt University. Through a series of trials, the osteotomy, repair and interfragmentary compression measurement processes were established.

### 2.2. Hole drilling and lateral condyle fracture model

**Figure 1:**
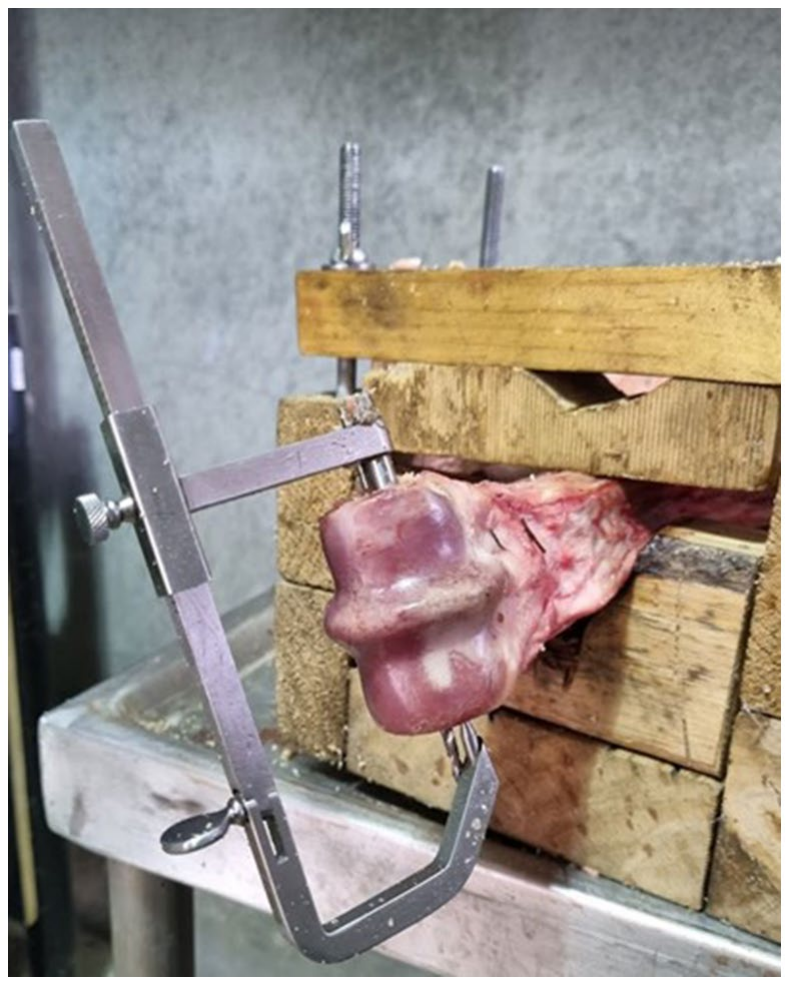
Aiming device in place with sample secured in a clamp to stabilize the bone for drilling.

**Figure 2:**
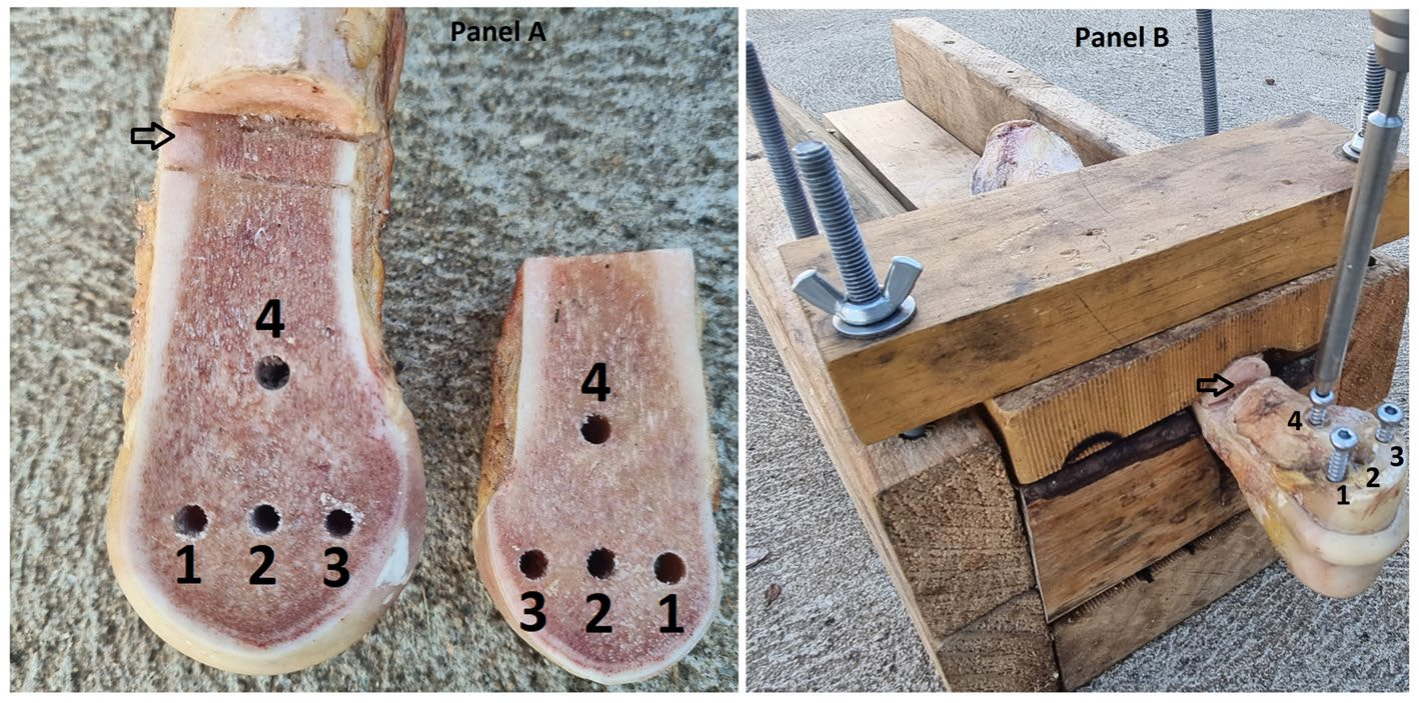
**(A)** Photograph of a left forelimb prepared with the opposing Cis and Trans osteotomy surfaces visible. The holes are numbered 1 = dorsal, 2 = central, 3 = plantar and 4 = proximal screw holes. The proximal osteotomy is indicated by an arrow. **(B)** The same sample with screws in holes 1, 3 and 4 for a triangular repair configuration. The image is labelled as for panel (A).

To prepare the samples for hole drilling, bones were thawed, in groups of four, at room temperature in a water bath. To facilitate ease of manipulation and consistent hole placement, the limbs were secured in a custom-made clamp (Figure 1). Following AO guidelines (17) for lag screw fixation, four drill holes were created in a triangular pattern. Holes have been numbered sequentially in a dorsal to palmar/plantar then proximal order, for reference purposes (Figure 2). All glide holes were drilled using an orthopedic aiming device, beginning with an initial 4.5 mm glide hole created in the center of the epicondylar fossa (hole 2). Three additional glide holes were placed. One dorsal (hole 1), one palmar (hole 3), and one 20 mm proximal (hole 4) to, the central hole. The holes created for triangular fixation were 10 mm from the dorsal and palmar/plantar articular surface of the bone. Glide holes were measured as 30 mm deep for the epicondyle and 10 mm deep for the proximal screw hole. The 3.2 mm drill sleeve was used to complete the 3.2 mm thread hole for each screw, and each thread hole was hand tapped using a 4.5 mm tap (vet Tap for Cortex Screws Ø 4.5 mm 311.460; DePuy Synthes Vet). All drilling and tapping procedures were performed with irrigation. After drilling and tapping, the limbs were wrapped in wet towels and re-frozen at −20°C.

To create the osteotomy in the frozen bones, an 800w, 200 mm table saw (TSB-0808, Ozito, Australia) fitted with a saw guide and 200 mm saw blade (200 mm 60 T Marathon, Irwin, Australia) was used. The osteotomy extended from the distal articular surface of the third metacarpal/metatarsal bone, adjacent to the sagittal ridge, and extended proximally for 75 mm. Two proximal osteotomies were performed, 10 mm apart, to complete the simulation of a complete fracture and allow for pressure-film placement and manipulation. Finally, the frozen bone pieces were secured together and wrapped with wet towels before being stored in a freezer at −20°C.

### 2.3. Repair technique and selection

Six limbs were utilized in preliminary investigations to fine tune the method of bone preparation, pressure film preparation, interfragmentary compression measurement and analysis. This left 28 limbs for investigation. Two screws, placed in screw holes two and four, were used in L and LW repairs. T and TD1 repair techniques employed three screws, placed in holes numbered one, three and four. Repairs were performed in a random order using a cross-over design. The initial repair selection approach was to perform L, LW, T TD1 repair techniques on each limb in a random order. Limbs were numbered one to twenty-eight, and each repair technique was assigned a number of one to four. A free source random number generator (random.org) (18) was used in two stages. The random sequence generator was used to create a random order of numbers between one and twenty-eight. Subsequently the random integer generator was used to select treatment method by generating 28 random integers between the numbers one and four, this process was repeated in case more than four repairs were possible per sample. Cadaver samples were used until screw stripping occurred to make use of cadaver samples. The order in which each repair technique was performed was recorded (Compression cycle), to determine the impact that repeated tightening and sample use had on the interfragmentary contact area measured.

### 2.4. Testing protocols

To prepare the samples for testing, bones were thawed in groups of four, at room temperature in a water bath. The osteotomy was cleared of debris and moisture to prevent artifacts on the pressure film. Screws were used in groups of five. When burring of the screwdriver seat was noted in one screw, all screws were replaced, resulting in a total of 25 screws being used.

### 2.5. Alternate screw tightening

Alternate tightening of 60 mm long, 4.5 mm cortical screws was performed for L, T, LW repairs. The order of screw insertion was dorsal (hole 1), palmar/plantar (hole 3) and proximal (hole 4) for T repairs. For L and LW repairs, the distocentral screw (hole 2) was placed prior to the proximal screw (hole 4; Figure 2). Each screw was tightened, by the primary author, until the screw head contacted the cortical bone or washer before moving on to the next screw. Subsequently, each screw was tightened to 4 Nm with the aid of a surgical screwdriver and quick-release torque limiter (511.771 Synthes, Raynham, MA, U.S.).

### 2.6. Sequential screw tightening

Sequential tightening of 60 mm long, 4.5 mm cortex screws was employed for TD1 repairs. The dorsal screw (hole 1) was tightened to 4 Nm, before the palmar/plantar (hole 3) and then, proximal (hole 4) screws were tightened to 4 Nm (Figure 2).

### 2.7. Interfragmentary compression measurement

To measure interfragmentary contact area and pressure, Prescale® pressure-sensitive film (Fuji Photo Film Co., Ltd., Japan) was used. The low-pressure (LLW) Prescale® film was determined through a preliminary experiment, as the most suitable option. The LLW film is 0.2 mm thick and can measure pressures ranging from 2.5 to 10 MPa. The pressure film is not validated for contact pressures of between 0 and <2.5 Mpa or of >10 and ≤12.5 MPa. All contact occurring at pressures of ≤12.5 MPA are recorded as 12.5 MPa. The pressure film consists of two layers labelled “A” and “C.” The “A” layer contains microcapsules that rupture at a specific threshold pressure, resulting in red areas following a reaction with the developer solution in the “C” layer. The color density changes according to the pressure exerted.

To prepare the Prescale® film for the experiment, the “A” and “C” layers were cut into 45 mm x 65 mm pieces and punched with holes, corresponding to the screw hole pattern, using a 6 mm paper hole punch. A paper template was used to transfer the hole pattern from each sample, and the lustrous surfaces of each film layer were placed in opposition to prevent any artefactual color leaching during the hole punching process. After punching four holes in each film, the film was correctly opposed and stapled together at a corner.

**Figure 3:**
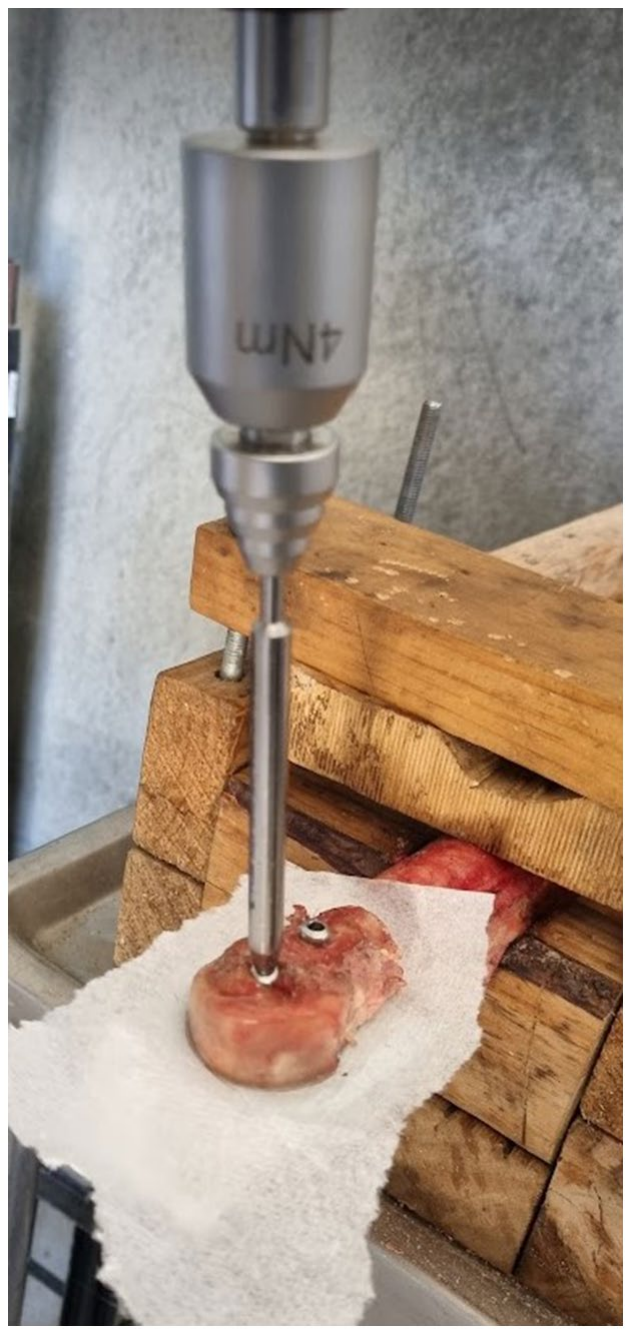
Picture of Linear configuration during simulated fracture repair, the paper towel is placed either side of the pressure sensitive film to prevent moisture artifacts.

The pressure film layers were then positioned in the osteotomy, inside two sheets of paper towel, and the initial repair configuration was placed before tracing the outer edge of the cis fragment using a pencil (Figure 3). Temperature and relative humidity readings were recorded, and screws were left in place for 2 min to measure static pressure, ensuring compliance with the recommended parameters outlined in the pre-scale manual. After screw removal, the pressure film was separated, and the “C” film was retained for analysis. This process was repeated for each repair technique, and any pressure film showing signs of moisture leaching or where screws stripped thread holes before reaching 4 Nm torque, were discarded. If a screw stripped before two prints were obtained from that limb, the limb was discarded and any data concerning that limb was removed from the study. All contact pressures of >10 MPa were grouped together, in accordance with the recommended threshold specifications of the pressure sensitive film.

### 2.8. Study design revision

Following implementation of the repair selection method in the first 16 limbs, four limbs were discarded for stripping of a thread hole, before two prints were obtained from that limb. Of the 12 limbs that remained in the study, it was possible to apply all four treatments in only four limbs, leaving a success rate of 4/16 limbs. After the high degree of sample dropout was encountered the repair selection process was revised. For the remaining 12 limbs, the L/T repair was randomly applied first, before moving to the LW/TD1 repairs. The order within each pair was determined by sequential digital coin toss (Random.org) (18).

### 2.9. Image processing

**Figure 4:**
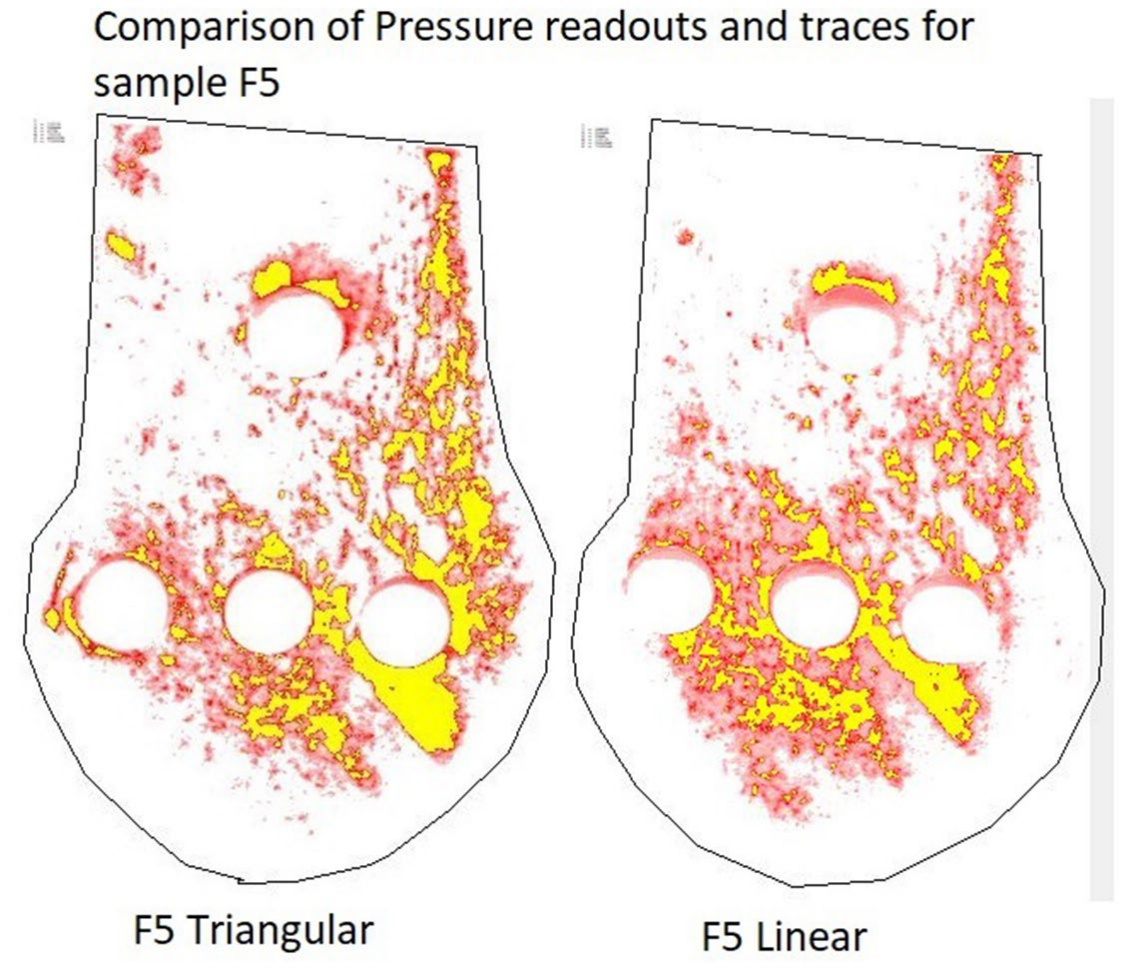
Screenshots of the same pressure film sample repaired with a linear and triangular configuration, processed with the Fujifilm software. Red areas correspond to contact area at pressures ≥2.5 MPa < 10 MPa. Yellow areas correspond to contact area at pressures >10 MPa. Pressures <2.5 MPa are below the working range of the pressure film, therefore have been removed.

The “C” film prints were trimmed along the pencil outline of the cis fragment. If minor variation occurred between samples following trimming, the largest of the prints was traced and recorded as the measured area, to ensure consistent size for the same sample. The samples were scanned with the recommended scanner and saved at a resolution of 96 Dots Per Inch (DPI) as JPEG images, before analysis with the provided software (Fujifilm Fuji FPD-8010E, Fuji Photo Film Co.). The software analyses the color density of each pixel which is calibrated based on the pressure threshold of the microcapsules in the pressure sensitive film. The results for total pressure area were categorized based on their values falling within the ranges of ≥2.5 MPA to ≤10 MPa, >2.5 MPa and > 10 MPa. Within the measurement threshold of the pressure sensitive film, further subcategories were created based on pressure values ranging from 2.5–3 MPa, 3–4 MPa, 4–5 MPa, 5–6Mpa, 6–7 MPa, 7–8 MPa, 8–9 MPa, and 9–10Mpa. All values of ≥10 MPa were grouped together and any values below 2.5 MPa were excluded from the final analyses because of the potential for artifacts caused by manipulation at low pressure readings (Figure 4).

### 2.10. Additional information

Prior to creation of the osteotomy fracture model, measurements of medial and lateral condylar width and diameter across the epicondylar fossa were obtained using a pair of Vernier calipers (ACCUD 300 mm IP67 Dual Scale Digital Vernier Caliper AC-112-012-12; Table 1).

**Table 1:** Mean measurements of cadaver bones used in experimental investigation.

Limb	Caudal width ± (sd)	Dorsal width ± (sd)	Medial condyle ± (sd)	Lateral condyle ± (sd)	Diameter lateral epicondyle ± (sd)	Diameter medial epicondyle ± (sd)
Front	58.6 mm ± (2.3)	51.5 mm ± (2.1)	29.4 mm ± (1.6)	26.2 mm ± (1.9)	34.1 mm ± (1.6)	35.3 mm ± (3.3)
Hind	59.2 mm ± (2.7)	51.4 mm ± (2.5)	29.7 mm ± (2.9)	27.2 mm ± (2.4)	35.0 mm ± (2.4)	39.2 mm ± (3.0)

### 2.11. Statistical analysis

Data were collected and imported into Microsoft Office Excel for further analysis. Descriptive and exploratory statistical analyses were performed using R software (19). To examine the interrelationships among the variables, Bayesian Network (BN) analysis was employed. The BN model was developed using the Netica software package (20). The descriptive data analysis aimed to provide a numerical summary of the sample data, while the BN model sought to quantify the impacts of the investigated influential factors through a what-if analysis. The results are expected to offer objective and quantitative assessment of the evidence supporting the establishment of generalizable research findings. It should be noted that the abbreviation s.d. is used to denote standard deviation. For the development of a BN model, it was technically necessary to discretize the continuous response variable data, a task that was automatically performed in Netica by dividing the range of the sample data into the most suitable intervals or categories. A BN model serves as a graphical representation of the joint probability distribution of all the variables included in the model. Nodes in the model represent the variables and are connected based on probabilistic dependencies (21). The BN approach is rooted in the mathematical formula of Bayes’ Theorem, which provides the theoretical foundation for this methodology. The formula is presented as Eq. 1 below (22).


(1) $$\mathrm{Pr}\left(B|A\right)=\mathrm{Pr}\left(A|B\right)\mathrm{Pr}\;\left(B\right)\;\mathrm{Pr}\;\left(A\right)=\mathrm{Pr}\;\left(A,B\right)\;\mathrm{Pr}\;\left(A\right)$$

Where A and B represent random variables, Pr*(A)* and Pr*(B)* denote the marginal probability distributions of A and B, respectively, Pr(B|A) represents the conditional probability distribution of B given A, Pr(A|B) represents the conditional probability of A given B, and Pr(A, B) is the joint probability distribution for A and B. Given that a BN model represents the joint probability of the variables, it allows for inferential analysis by fixing the values of a selected set of variables and predicting or estimating the values of the remaining variables in the model (23). Furthermore, the BN model offers several additional advantages, such as generating estimates and credible intervals for derived parameters or predicted variables. Moreover, it allows for the quantification of support in favor of the null hypothesis, not just against it (24).

A BN model consists of two components: qualitative and quantitative. The qualitative component specifies the network structure by connecting all variables/nodes in the model, while the quantitative component determines the conditional probability tables that quantify the strengths of dependence relations using probability theory (21, 23, 25). In this study, the BN model was developed by determining the optimal model structure using the Tree Augmented Naïve-Bayes-Net (TAN) algorithm, and the model parameters were estimated using the Expectation–Maximization (EM) algorithm in Netica (23). The prediction performance of the resulting BN model was evaluated and compared with the sample data set using Netica’s “Testing a Net Using Cases” function. Netica also includes the “Sensitivity to Findings function,” which allows ranking of impacts of other variables (referred to as “evidence variables”) on a selected target variable (23).

**Figure 5:**
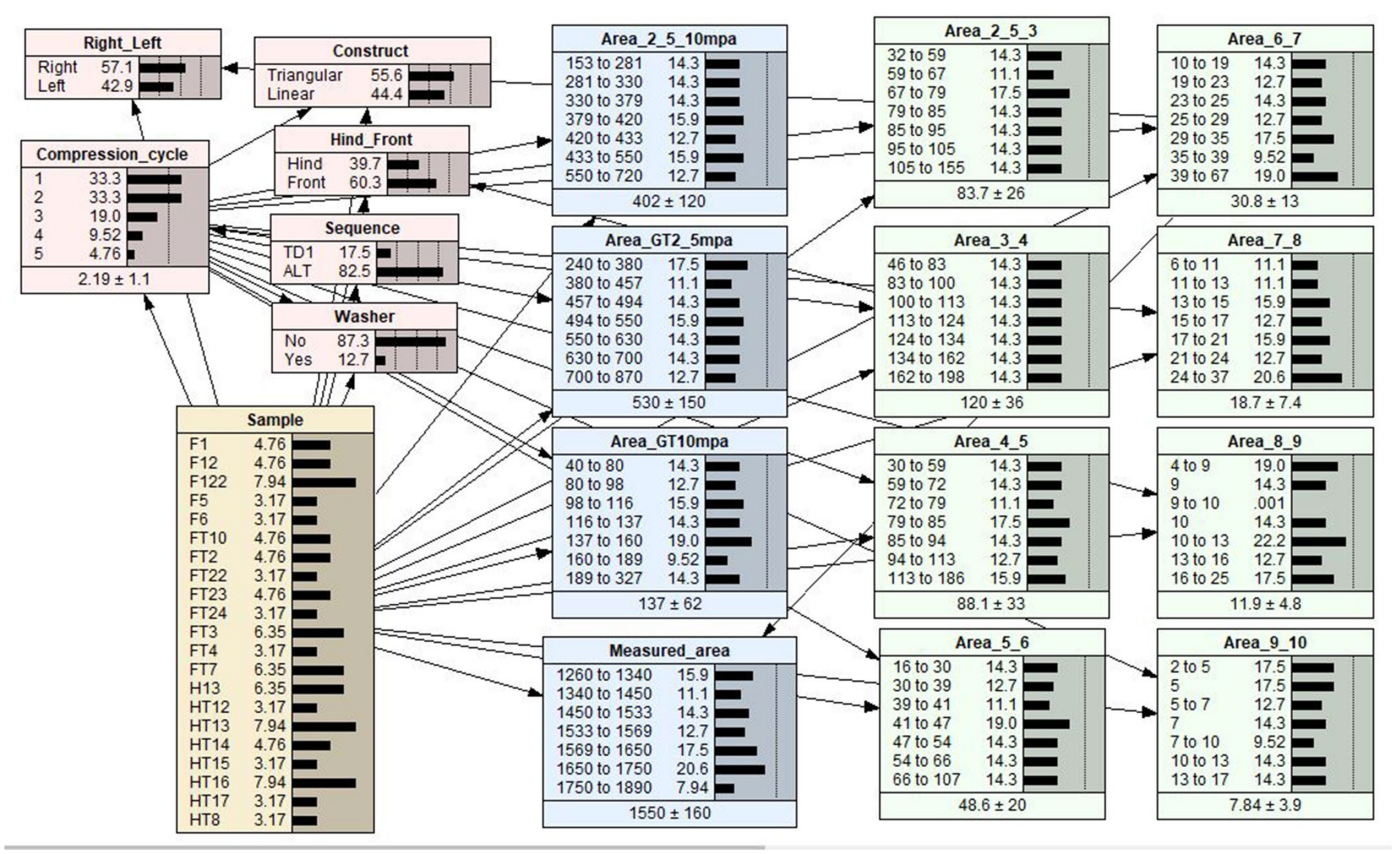
A Bayesian network (BN) model of the relationships between effect variables and contact area. Each variable in the BN model is represented by a node. The link between two nodes represents the dependency relationship between two variables. The middle column of each node is a percentage totaling to 100%, which represents the analysis outcomes of each level within a node. The last column is a graphical representation of the percentage values for each level shown as distribution bars. The dotted lines are markers that are equally spaced to aid in visualizing the comparative heights of the distribution bars.

To investigate the most influential variable in determining contact area under pressure, a BN model was constructed by considering the variables Construct (L, T, LW, TD1), Limb (front, hind), Limb (right, left), Compression cycle and Measured area (Cis fragment total area; Figure 5) By selecting different potential variables, the BN model estimated or predicted the mean contact pressure outcomes for L, T, TD1, and LW repairs (Table 2). A table documenting the mean contact areas predicted by the BN model is included as (Supplementary Table 2).

**Table 2:** Mean contact area at all pressures above the minimum working threshold of the pressure sensitive film.

Treatment	Contact area ≥ 2.5 MPa ≤10 MPa (sd.)	Contact area > 10 MPa (sd.)	≥ 2 5 Mpa	≥ 2.5 Mpa ≤3 Mpa	≥ 3 Mpa ≤4 Mpa	≥ 4 Mpa ≤5 Mpa	≥ 5 Mpa ≤6 Mpa	≥ 6 Mpa ≤7 Mpa	≥ 7 Mpa ≤8 Mpa	≥ 8 Mpa ≤9 Mpa	≥ 9 Mpa ≤10 Mpa
Linear (L)	403 mm^2^ ± (140)	112 mm^2^ ± (48)	517 mm^2^ ± (152)	86 mm^2^ ± (26)	86 mm^2^ ± (26)	87 mm^2^ ± (33)	46 mm^2^ ± (19)	28 mm^2^ ± (12)	17 mm^2^ ± (7)	11 mm^2^ ± (4)	7 mm^2^ ± (4)
Triangular (T)	411 mm^2^ ± (120)	167 mm^2^ ± (70)	573 mm^2^ ± (156)	80 mm^2^ ± (24)	119 mm^2^ ± (34)	87 mm^2^ ± (25)	49 mm^2^ ± (15)	32 mm^2^ ± (10)	20 mm^2^ ± (7)	13 mm^2^ ± (5)	9 mm^2^ ± (4)
Linear + washer (LW)	366 mm^2^ ± (70)	100 mm^2^ ± (27)	465 mm^2^ ± (64)	81 mm^2^ ± (18)	86 mm^2^ ± (26)	77 mm^2^ ± (14)	41 mm^2^ ± (9)	25 mm^2^ ± (5)	14 mm^2^ ± (3)	10 mm^2^ ± (2)	5 mm^2^ ± (1)
Triangular, sequential tightening (TD1)	403 mm^2^ ± (120)	142 mm^2^ ± (50)	526 mm^2^ ± (150)	79 mm^2^ ± (23)	113 mm^2^ ± (36)	80 mm^2^ ± (26)	45 mm^2^ ± (16)	28 mm^2^ ± (11)	17 mm^2^ ± (7)	12 mm^2^ ± (5)	8 mm^2^ ± (3)

Contact areas were calculated fixing the relevant nodes in the BN model. For example in the linear repair investigation. The Construct, sequence and washer nodes were fixed as Linear, Sequential, and No, respectively.

A sensitivity analysis was performed for contact area (the target variable) to quantify the strength of the association between the interrelated variables. Netica inherently includes the *sensitivity to findings* function, allowing for the ranking of impacts of other variables (referred to as “evidence variables”) on a selected target variable (23). The outcomes of the sensitivity analysis, for all pressure categories, are presented in Table 3. These sensitivity outcomes represent the strength of association between the evidence variables and the target variable (20). The percentage values obtained from the sensitivity analyses for a selected target variable are broadly analogous to the adjusted R^2^, a goodness-of-fit measure used in regression analysis that represents the proportion of the variation explained in the selected variable by fixing the value in one of the evidence variables.

**Table 3:** Bayesian network sensitivity findings indicating the effect each variable had on the contact area at respective pressure intervals.

Node	Sensitivity findings (reduction of variance) of the BN model in percent (%). In each pressure category for the respective variable. Percentages given to three significant figures.										
	≥ 2.5 Mpa ≤10 Mpa	> 10 Mpa	≥ 2.5 Mpa	≥ 2.5 Mpa ≤3 Mpa	≥ 3 Mpa ≤4Mpa	≥ 4 Mpa ≤5 Mpa	≥ 5 Mpa ≤6 Mpa	≥ 6 Mpa ≤7 Mpa	≥ 7 Mpa ≤8 Mpa	≥ 8 Mpa ≤9 Mpa	≥ 9 Mpa ≤10 Mpa
Limb (right/left)	5.79	23.3	14.7	0.623	2.13	7.54	7.8	10.2	12.2	8.52	14.9
Construct (T,L)	0.414	16.3	6.97	1.56	0.00518	0.019	3.37	4.11	5.81	9.42	9.06
Measured Area	30.6	12.8	19.1	30.4	29	28.4	20.6	22.1	18.1	18.9	22.5
Washer	1.18	5.03	4.02	0.0171	0.704	1.43	1.38	1.96	4.51	3.01	7.13
Limb (hind/front)	2.38	2.98	0.386	6.43	2.91	0.998	1.22	0.386	0.65	0.63	0.766
Compression cycle	2.84	2.04	3.49	4.69	3.22	3.65	3.11	3.64	1.57	2.71	0.717
Sequence (Alternate/ TD1)	0.00172	0.133	0.519	0.000657	0.342	0.354	0.0776	0.268	0.017	2.02	0.00645

Sensitivity findings are the percentage reduction of variance. Data is displayed to three significant figures. Shaded area represents the most influential node in each category. Each node corresponds with the nodes of the BN model (Figure 7).

## 3. Results

### 3.1. Limbs and repairs

**Figure 6:**
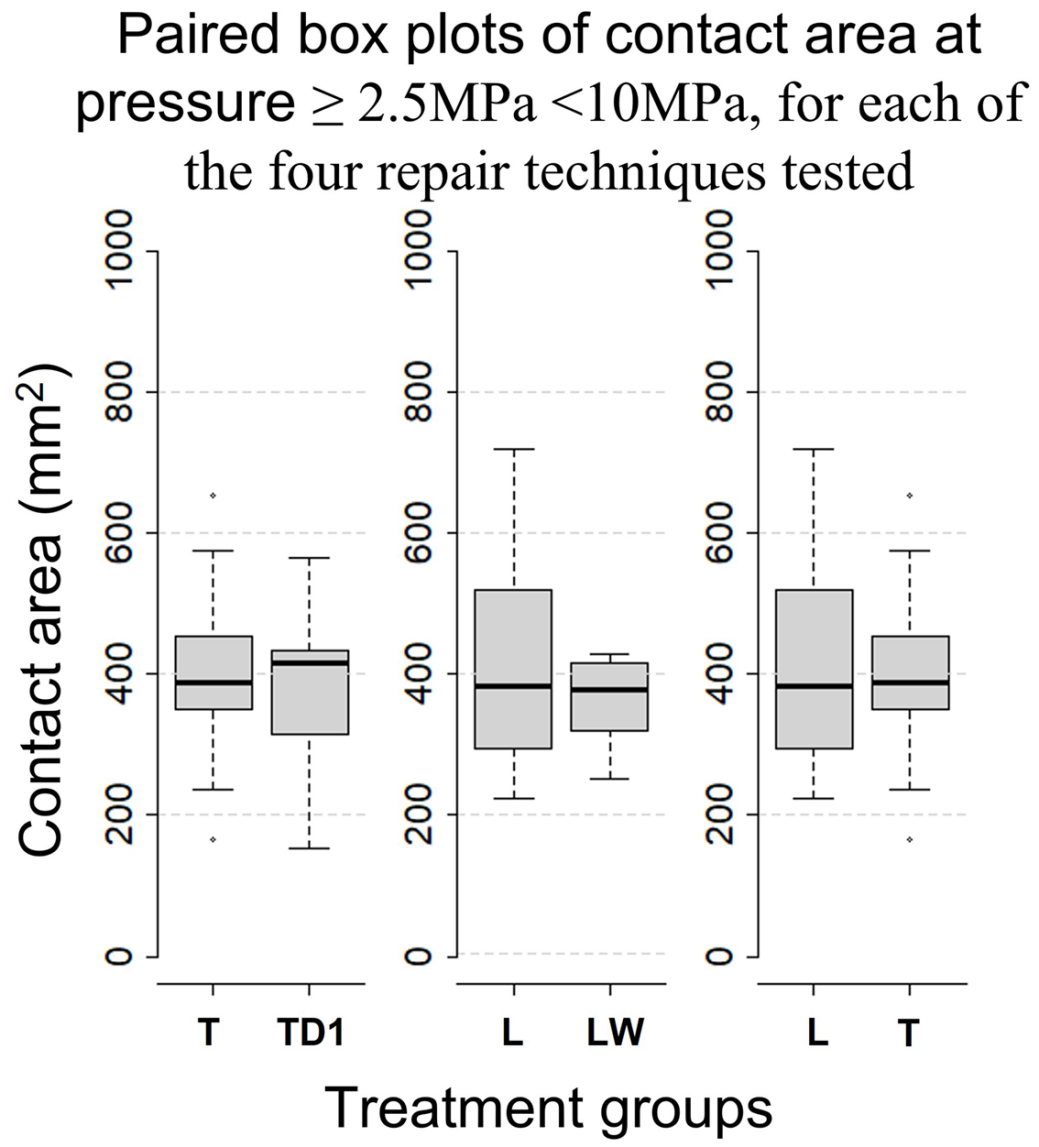
Paired Box-whisker plots comparing contact area for pressures of ≥2.5 MPa < 10 MPa between each repair configuration, calculated during descriptive analysis. Box-whisker plots compare the distributions of sample data. Namely, comprehensive comparisons can be made in terms of medians (the bold horizontal bars), the boxes (the middle 50% data points), the minima and maxima, and the extreme cases (outliers) that exceed the 1.5 inter-quartile range (the range of the box).

**Figure 7:**
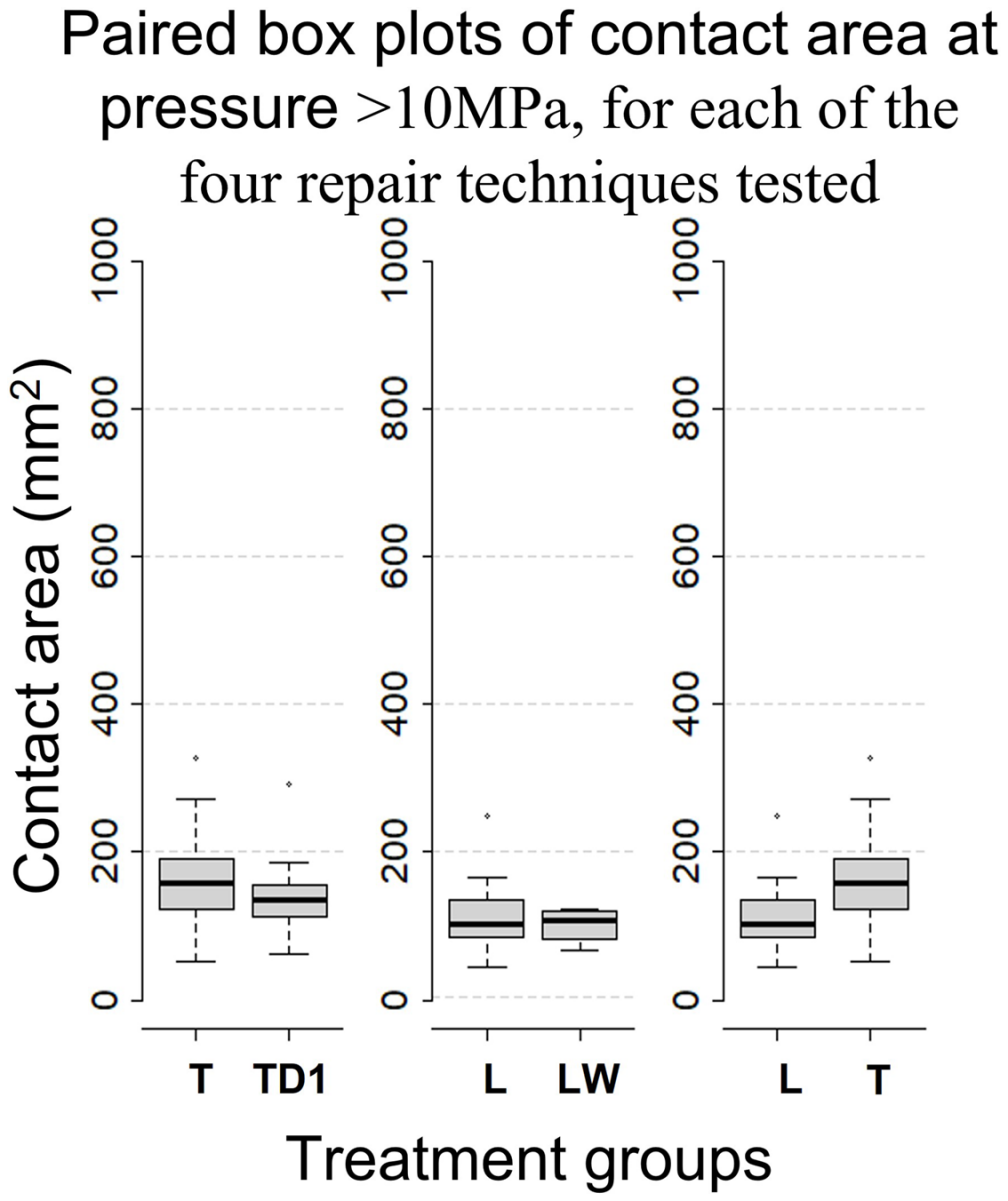
Box-whisker plots, calculated during descriptive analysis, comparing mean contact area between repair configurations for pressure > 10Mpa.

28 limbs obtained from horses of mixed breed underwent fracture simulation and repair. Weight and sex were unknown. Thread holes stripped following the first repair in six limbs and one screw broke through the articular surface during tightening in a further limb. Resulting in a total of Sixty-three repairs (20*L, 24* T, 11*TD1, and 8*LW) performed on 21 (13 front, 8 hind) limbs being included in the analysis. Screw hole stripping occurred most often at the proximal screw hole (18 times), followed by 6 times at the palmar/plantar screw hole and twice each at the central and dorsal screw holes. Average anatomical measurements of the samples included in the final analysis can be found in Table 1. The mean contact area, calculated during descriptive analysis (±s.d.), created at pressures within the operating range of the Prescale® film (≥ 2.5 MPa ≤ 10 MPa) by L, T, TD1 and LW repairs were 406 mm^2^ ± (132), 404 mm^2^ ± (112), 382 mm^2^ ± (119), and 363 mm^2^ ± (63) respectively. The mean contact area (±s.d.) created by L, T, TD1 and LW repairs at pressures >10 MPa were 110 mm^2^ ± (45), 164 mm^2^ ± (67), 144 mm^2^ ± (59), and 101 mm^2^ ± (23) respectively (Table 2). Median contact areas were greater for T repairs when compared to L repairs at pressures ≥2.5 MPa ≤ 10 MPa and >10 MPa. Median contact areas were greater for L than LW repairs at pressures ≥2.5 MPa ≤ 10 MPa, however at pressures >10 MPa median contact area of LW repairs was greater than L repairs. The median contact areas for T repairs were greater than TD1 repairs at pressures in the >10 MPa category, however at pressures ≥2.5 MPa ≤ 10 MPa the median contact area of TD1 repairs was larger than T repairs (Figures 6, 7). A complete breakdown of mean and median contact areas, calculated during descriptive analysis is available in the (Supplementary Table 1).

### 3.2. Bayesian network model

The goodness-of-fit of the BN model was evaluated using the built-in Netica function *Test with Cases*. The assessment focused on the target variable of contact area in the ≥2.5 Mpa ≤ 10, >10 MPa pressure categories. The model exhibited an error rate of 0% for the ≥2.5 Mpa ≤ 10 and >10 MPa categories.

The sensitivity analysis outlined in Table 3, show that measured area had the strongest model predicted relationship (sensitivity finding of 30.6%) with mean contact area in the ≥2.5 Mpa ≤ 10 Mpa pressure category whereas limb (right or left) had the strongest predicted relationship (sensitivity finding of 23.36%) in the >10 MPa pressure category. The BN model predicted that T repairs resulted in the largest mean contact area in the ≥2.5 Mpa ≤ 10 Mpa and >10 MPa pressure categories. The sensitivity analysis indicated that repair configuration had a weak influence (0.414%) in the ≥2.5 Mpa ≤ 10 Mpa category and a moderate influence (16.3%) in the >10 MPa pressure category on mean interfragmentary contact area. In the ≥2.5 Mpa ≤ 10 Mpa and >10 MPa pressure categories the addition of a washer to a linear configuration explains 1.18and 5.03% of the difference in mean interfragmentary contact area, respectively. The sensitivity analysis indicated that less than 1% of the variation in mean interfragmentary contact area can be attributed to the TD1 repair configuration, indicating a weak influence of sequential tightening on mean interfragmentary compression. The sensitivity analysis indicated repeated use of the same sample (compression cycle) and whether the sample tested was a hind or forelimb had a weak effect on contact area produced (Table 3).

## 4. Discussion

In this study, the investigation focused on interfragmentary compression achieved by various constructs in the repair of simulated lateral condylar fractures. The BN model showed that T repairs produced a greater mean contact area compared to L repairs. This difference was greatest at pressures greater than10 MPa. Sensitivity analysis indicated that 16.1% of the variation in contact area was attributed to the construct at pressures > 10Mpa. The effect of construct on interfragmentary compression in this pressure range is considered moderate. In comparison, 0.414% of the variation in contact area at pressures ≥2.5 Mpa ≤ 10 Mpa could be attributed to the construct. These findings suggest that triangular constructs create larger contact areas at higher pressures compared to linear constructs.

The sensitivity analysis findings presented in Table 3, indicated that the measured area exhibited the highest sensitivity index (30.6%) at pressures between 2.5 and 10 Mpa. This result was expected as the larger surface areas of fragments provide a greater overall area to exert pressure. Additionally, the reduced impact of the measured surface area (sensitivity finding of 12.8%) in the greater than 10 Mpa category aligns with the fact that lag screws have the greatest effect adjacent to the screw (5). Variation in bone size is an obvious contributor to the variation in measured area. However, as the bones were noted to be of similar size (Table 1), the variation in measured area is likely further influenced by the orientation and location of the osteotomy cut. Despite efforts to avoid variation in osteotomy location, because of the tapered anatomy of the condyle, even slight variation in the osteotomy location can result in an appreciable difference in surface area between samples. The limb (right or left) exhibited the highest sensitivity index at pressures greater than 10 Mpa, which can also be attributed to variation in the measured area.

Interfragmentary compression is an essential element of lag screw fixation, relying on interfragmentary friction to maintain stability across a fracture (5, 26). The results of this study indicate that a T repair creates greater interfragmentary compression than a L repair at higher pressures. Greater interfragmentary compression, as demonstrated in T repairs, may contribute to a more stable repair. Although, interfragmentary compression resulting from linear or triangular repairs of the proximal phalanx has not been assessed, increased interfragmentary compression may contribute to the advantages of T repair over L repair, as observed in a previous experimental model investigating proximal P1 repairs (14). Unlike this study, the aforementioned study assessed triangular and linear repair in maintaining fracture stability in loaded limbs (14). *In vivo*, torque placed on the limb has a greater effect on fracture stability than the force placed perpendicular to the bone’s long axis (27). As compression achieved by placement of a lag screw effects only a small region of adjacent bone, the cumulative interfragmentary friction created by an additional screw in T repairs may contribute to their advantages over L repairs under load (5, 14).

In this investigation, the use of a washer was found to reduce the overall mean interfragmentary contact area at pressures of ≥2.5 Mpa ≤ 10 Mpa and >10 Mpa when comparing linear constructs with and without washers. However, the sensitivity analysis indicated that the influence of using a washer on contact area was minor, with findings of 1.08 and 5.11% for the respective ≥2.5 Mpa ≤ 10 Mpa and > 10 Mpa pressure categories. Previous studies have suggested that washers can improve interfragmentary compression by reducing screw intrusion into the cortical bone, allowing for greater screw torque before intrusion occurs (28). However, in this investigation, the torque applied to each construct was uniform, and therefore the advantage of using a washer was not apparent. It has been suggested that a larger screw head may result in greater stability compared to a standard 8 mm lag screw head, even without an increase in measurable interfragmentary compression (12). The size of the washer used in this study was the same diameter as the modified screw head (10 mm) used in that previous study. However, it is important to note that the use of a washer cannot be directly compared to the use of a larger screw head since the washer reduces friction between the screw head and the cortical bone interface, while a larger screw head increases friction at this location (12). This study indicates that that addition of a washer does not improve interfragmentary compression, without increased screw torque.

Results of this investigation showed that triangular repairs tightened alternately (T), on average, created greater contact area than sequentially tightened triangular repairs (TD1). However, the sensitivity analysis indicated that tightening sequence has a very weak influence on interfragmentary compression. These results, do not align with reports suggesting that alternate tightening of two lag-screws can reduce fracture displacement during fixation and distribute even compression across the fracture surface (16). When considering engineering applications, uneven tightening of bolted joints can result in bolt cross-talk, where tightening one bolt decreases the preload in another previously tightened bolt (29). The similarity between T and TD1 contact area prints, suggests that tightening the initial screw, did not prevent the second screw from producing comparable contact pressure to those constructs where alternate tightening was employed. It should be noted that the measurement technique used in this investigation assessed peak pressure, so it is possible that changes in pressure around the initial screw occurred during the tightening process but were not observed. In practice, despite no marked advantages with regards to interfragmentary compression, alternate tightening may allow for even fracture gap reduction (16).

In this study, a screw insertion torque of 4 Nm was used. But, the optimum lag screw insertion torque is not known (12). It should be noted that 4 Nm is less than the perceived maximum torque employed by experienced equine surgeons, as demonstrated in previous research (12). Countersinking was not performed at any of the screw insertion sites in this study, due to spatial limitations within the epicondylar fossa. Opinions on the clinical use of countersinking in the distal-most screw placement differ among surgeons. Some advocate for countersinking in this location, while others argue that the concave structure of the epicondyle allows for satisfactory apposition of the screw head, and countersinking may result in damage to the collateral ligament. Unlike in areas of thin cortical bone such as the proximal phalanx, countersinking contributing to cortical bone failure beneath the screw head is unlikely to occur in the dense bone of the epicondyle (2, 30). In general countersinking improves contact at the screw bone interface and improves interfragmentary compression (2). It is important to emphasize that the primary aim of this investigation was to compare the effect of various treatments on interfragmentary compression, rather than assessing maximum interfragmentary compression. The choice of 4 Nm was based on its frequent use in research and the availability of a recognized orthopedic torque limiter for this value (12). The consistent screw torque placed and the absence of countersinking on all screws allowed an accurate comparison between construct configurations. Investigating the interfragmentary compression achieved by maximal perceived torque and with the use of countersinking, are an obvious target for future investigations.

Various locations have been suggested as the optimal position for the distal screw when repairing condylar fractures. Some authors have recommended placing the distal screw at the level of the epicondylar tubercle to prevent collateral ligament damage, while others proposed placing the screws as close to the articular surface as possible (11). Currently, the recommended location for screw placement is at the center of the epicondylar fossa (10, 26). From a general orthopedic perspective, there is no recommended minimum distance between a screw and the edge of a bone or joint surface. However, in industrial applications, it is recommended to place a lag screw no closer than 1.25 times the screw’s diameter to the edge of the material being fastened (31). In orthopedic applications, neighboring screws should be placed no closer than twice the diameter of the screw being used and remain at least one diameter of the screw from a fracture line (32). The epicondylar fossa permits little room for variation in screw placement (2) making triangular repair more difficult when compared to linear repair. In this investigation screws were placed 10 mm from the dorsal and palmar/plantar articular surfaces of the condyle. However, even with the use of an aiming device and the luxury of placing screws in the absence of soft tissue, one screw broke into the articular surface. Placing screws closer than 11.9 (±3.9 mm) to the articular surface of the fetlock joint during condylar fracture repair can result in a reduced rate of successful return to racing (11, 33, 34). In contrast, when repairing fractures of the proximal phalanx, it is recommended to place screws between 5 and 8 mm from the distal extent of the sagittal groove. To date, reduced success rates and complications arising from close placement of implants to the articular surface of the proximal phalanx have not been reported (35). Spatial limitations dictate that meticulous planning, and the use of intraoperative imaging should be considered essential if placing parallel screws in the distal aspect of the third metacarpal or metatarsal bone and the possible negative effects of placing screws closer than 10 mm to the joint surface should be considered.

The cadaver samples used in this investigation were sourced from mixed breed horses euthanized for reasons unrelated to the study. The weight, breed, and sex of the horses used in this study were not available, but the measurements presented in Table 1, indicate that the bones were similar in size. Although a large proportion of the study population was likely to be racehorses, the lack of information regarding cadaver samples should be accounted for when considering generalization of the results of this study.

To ensure bicortical engagement, 60 mm screws were selected for this investigation. The tapered anatomy of the condyle, documented by the anatomical measurements in Table 1, dictates different screw lengths are required for parallel screw placement. A longer palmar/plantar screw will be necessary when compared to the dorsal screw, whereas the dorsal screw length approximates the screw length required in centrally placed distal screws of linear repairs.

The initial study design was deemed unrealistic because of the significant loss of samples caused by thread hole stripping. Thread hole stripping predominantly occurred at the proximal screw hole (hole 4), which was utilized for all constructs. Moreover, the cortical bone at this location is thinner and has a lower density compared to the corresponding bone within the epicondyle region (2, 36). To achieve the cleanest possible cut, following a trial-and-error approach, a table saw was employed in frozen bones. To mitigate the impact of surgical technique on fracture reduction and subsequent interfragmentary compression, holes were drilled prior to creating the osteotomy. Consequently, the bones had to be thawed for drilling, before being re-frozen for the osteotomy. The osteotomy procedure could be performed swiftly, and all bones were returned to the freezer before any noticeable thawing occurred. Despite wrapping the bones in moist towels, thawing in small groups, and keeping the bones moist while handling and drilling, the repeated freeze–thaw process may have contributed to the high occurrences of thread hole stripping. However, it must be noted that it was possible to place at least four repairs in six of twenty-one cadaver specimens. Ideally, drilling holes in fresh samples before freezing them in preparation for the osteotomy and fixation would have eliminated the need for multiple thaws. However, logistical constraints meant this approach was not feasible. Nonetheless, the tendency for thread holes to strip at the proximal screw hole underscores the crucial role of precise surgical technique to avoid unnecessary repetition. It must be acknowledged that changes to the bone were likely to occur as a result of the repeated use of the same sample. However, repetition fatigue through use of the same sample was shown to have only a weak influence on the interfragmentary contact area, as shown by the sensitivity findings of the BN model (Table 3).

In this study, Screws were used, until burring of the screw-driver seat was noted. This resulted in screws being used an inconsistent number of times. Clinically, it is not recommended to reuse screws. The effect of repeated tightening of screws through the bone samples on interfragmentary compression was considered weak, however the effect of multiple uses of each screw was not analyzed. The inconsistency in screw cycle usage should be taken into account when considering the results of this study. The repeated use of screws may have contributed to the high rate of thread hole stripping noted in this study. However, as each screw was tightened to a consistent torque the interfragmentary compression is relative for each repair and the results of this study should be considered valid.

In the present study, interfragmentary compression was evaluated using Prescale® film, a pressure measurement system that has been employed in various veterinary orthopedic investigations (37, 38). Prescale® film was chosen for its flexibility, thinness, and capacity to be cut into different shapes (39). The Low pressure (LLW) pressure film utilized in this study operates in the range of 2.5–10 MPa. When comparing each construct, we considered the area pressed, as it provided the most precise measurement that enabled comparisons in this scenario. Load was unable to be calculated in this investigation, because, once force surpassed the upper threshold of the pressure-sensitive film (10 MPa), the accuracy of the film diminished, and all values above 12.5 MPa were recorded as 12.75 MPa (40). Grouping all pressures above 12.75 MPa prevented the precise calculation and comparison of load between constructs. Based on the results of this study, we determined that a single Prescale® film that can assess all pressures exerted across the osteotomy during fixation is not available. Using a dynamic digital film pressure measurement system would have allowed for the evaluation of the entire range of contact pressures and facilitated the assessment of any dynamic reduction in preload during the sequential tightening experiment. However, the digital sensor technology is expensive, and punching holes in the digital film sensor can be challenging and cause sensor damage (41). The cost of the digital pressure mapping system was beyond the financial capacity of this investigation.

## 5. Conclusion

This investigation assessed the interrelationships between several different fixation techniques and interfragmentary compression, when repairing simulated lateral condylar fractures. Moderate evidence was found to suggest that triangular repairs result in greater interfragmentary compression when compared to linear repairs. Employing a sequential tightening sequence and the addition of a washer resulted in reduced mean interfragmentary compression when compared to alternate screw tightening and a linear repair without a washer, respectively. However, the impact of these factors can be considered weak. The greater interfragmentary compression provided by triangular repairs, may provide greater stability in lateral condylar fracture repair. The increased surgical difficulty and possible impacts of placing screws near the articular surface should be kept in mind when considering the potential clinical advantages of triangular repair. This study provides a valuable piece of evidence towards building a generalized understanding of interfragmentary compression achieved with triangular repair. However, the previously stated study limitations must be considered and further research is required to evaluate the effect of triangular repair in clinical cases.

## Data availability statement

The raw data supporting the conclusions of this article will be made available by the authors, without undue reservation.

## Ethics statement

The animal study was approved by Animal Care and Ethics Charles Sturt University. The study was conducted in accordance with the local legislation and institutional requirements.

## Author contributions

AB designed the study and wrote the first draft of the manuscript. KH and RL contributed to the review of the study design and manuscript and approve the submitted version.

## Funding

AB was supported by an Australian Research Training Program Scholarship from Charles Sturt University.

## Conflict of interest

The authors declare that the research was conducted in the absence of any commercial or financial relationships that could be construed as a potential conflict of interest.

## Publisher’s note

All claims expressed in this article are solely those of the authors and do not necessarily represent those of their affiliated organizations, or those of the publisher, the editors and the reviewers. Any product that may be evaluated in this article, or claim that may be made by its manufacturer, is not guaranteed or endorsed by the publisher.
