# Supplementary material for: Comparison of interfragmentary compression across simulated condylar fractures repaired using four techniques
Source: Front Vet Sci. 2023 Sep 20;10:1233921. doi: 10.3389/fvets.2023.1233921 (PMC10553202; doi:10.3389/fvets.2023.1233921)
Supplement: Supplementary file 1 [file Table_1.pdf]

Supplementary Material

Comparison of interfragmentary compression across simulated condylar fractures repaired using four techniques

Ashley Brabon*, Kristopher Hughes, Raphael Labens

*** Correspondence:** Ashley Brabon: abrabon@csu.edu.au

1 Supplementary Tables

Supplementary Table 1 Comparison of mean and median contact area at all pressures above the minimum working threshold of the pressure sensitive film. Contact areas were calculated during descriptive analysis in software R.

**Treatment**	Contact area as derived from descriptive analysis (sd)									
	≥ 2.5 Mpa ≤ 10 Mpa	> 10 Mpa	≥ 2.5 Mpa	≥ 2.5 Mpa ≤ 3 Mpa	≥ 3 Mpa ≤ 4Mpa	≥ 4 Mpa ≤ 5 Mpa	≥ 5 Mpa ≤ 6 Mpa	≥ 6 Mpa ≤ 7 Mpa	≥ 7 Mpa ≤ 8 Mpa	≥ 8 Mpa ≤ 9 Mpa	≥ 9 Mpa ≤ 10 Mpa
**Mean Linear (L)**	406 mm^2 ± (132)	110 mm^2 ± (45)	517 mm^2 ± (152)	87 mm^2 ± (26)	124 mm^2 ± (40)	87 mm^2 ± (33)	46 mm^2 ± (19)	28 mm^2 ± (12)	17 mm^2 ± (7)	11 mm^2 ± (4)	7 mm^2 ± (4)
**Median Linear (L)**	382 mm^2 ± (132)	103 mm^2 ± (45)	488 mm^2 ± (152)	83 mm^2 ± (26)	118 mm^2 ± (40)	79 mm^2 ± (33)	42 mm^2 ± (19)	24 mm^2 ± (12)	16 mm^2 ± (7)	10 mm^2 ± (4)	6 mm^2 ± (4)
**Mean Triangular (T)**	404 mm^2 ± (112)	164 mm^2 ± (67)	573 mm^2 ± (156)	80 mm^2 ± (24)	119 mm^2 ± (34)	87 mm^2 ± (25)	49 mm^2 ± (15)	32 mm^2 ± (10)	20 mm^2 ± (7)	13 mm^2 ± (5)	9 mm^2 ± (4)
**Median Triangular (T)**	388 mm^2 ± (112)	158 mm^2 ± (67)	546 mm^2 ± (156)	78 mm^2 ± (24)	121 mm^2 ± (34)	85 mm^2 ± (25)	48 mm^2 ± (15)	30 mm^2 ± (10)	18 mm^2 ± (7)	12 mm^2 ± (5)	7 mm^2 ± (4)
**Mean Linear +**	363 mm^2 ± (63)	101 mm^2 ± (23)	465 mm^2 ± (64)	81 mm^2 ± (18)	110 mm^2 ± (20)	77 mm^2 ± (14)	41 mm^2 ± (9)	25 mm^2 ± (5)	14 mm^2 ± (3)	10 mm^2 ± (2)	5 mm^2 ± (1)

**washer (LW)**											
**Median Linear + washer (LW)**	379 mm^2 ± (63)	108 mm^2 ± (23)	482 mm^2 ± (64)	80 mm^2 ± (18)	114 mm^2 ± (20)	79 mm^2 ± (14)	41 mm^2 ± (9)	24 mm^2 ± (5)	13 mm^2 ± (3)	10 mm^2 ± (2)	5 mm^2 ± (1)
**Mean Triangular dorsal screw tightened first (TD1)**	382 mm^2 ± (119)	144 mm^2 ± (59)	526 mm^2 ± (150)	79 mm^2 ± (23)	113 mm^2 ± (36)	80 mm^2 ± (26)	45 mm^2 ± (16)	28 mm^2 ± (11)	17 mm^2 ± (7)	12 mm^2 ± (5)	8 mm^2 ± (3)
**Median Triangular dorsal screw tightened first (TD1)**	415 mm^2 ± (119)	136 mm^2 ± (59)	550 mm^2 ± (150)	81 mm^2 ± (23)	116 mm^2 ± (36)	81 mm^2 ± (26)	45 mm^2 ± (16)	29 mm^2 ± (11)	19 mm^2 ± (7)	11 mm^2 ± (5)	8 mm^2 ± (3)

Supplementary Table 2 The Mean contact area for each dependent variable and all possible configurations modelled in the BN model.

Variables						Mean contact area mm^2 ± (SD), by pressure category										
**Limb (right/left)**	**Construct (L/T)**	**Limb (front/hind)**	**Sequence (Seq/TD1)**	**Washer (yes/no)**	**Mean measured area mm^2**	**≥ 2.5 Mpa ≤ 10 Mpa**	**> 10 Mpa**	**≥ 2.5 Mpa**	**≥ 2.5 Mpa ≤ 3 Mpa**	**≥ 3 Mpa ≤ 4Mpa**	**≥ 4 Mpa ≤ 5 Mpa**	**≥ 5 Mpa ≤ 6 Mpa**	**≥ 6 Mpa ≤ 7 Mpa**	**≥ 7 Mpa ≤ 8 Mpa**	**≥ 8 Mpa ≤ 9 Mpa**	**≥ 9 Mpa ≤ 10 Mpa**
Right	T	Hind	Seq	No	No	476 ± (160)	258 ± (40)	675 ± (120)	83.2 ± (17)	136 ± (46)	107 ± (42)	61.7 ± (22)	39.2 ± (13)	25.6 ± (7.2)	15.4 ± (4.7)	12.5 ± (3.7)
Right	T	Hind	TD1	No	1600± (120)	439 ± (44)	186 ± (59)	655 ± (98)	87.3 ± (4.6)	125.5 ± (5.8)	91.7 ± (9.6)	51.5 ± (7.0)	39 ± (11)	21.3 ± (1.9)	14.5 ± (4.6)	11.7 ± (2.8)
Right	L	Hind	Seq	No	1660 ± (120)	397 ±	124 ± (21)	532 ±	78.6 ± (33)	121 ± (53)	99.7 ±	50.5 ± (25)	31.2 ± (15)	19.2 ± (8.2)	11.0 ± (5.4)	9 ± (4.8)

						(170)		(200)			(45)					
Right	L	Hind	Seq	yes	1620 ± (110)	390 ± (37)	126.5 ± (6.1)	499 ± (27)	82 ± (1.8)	112.5 ± (6.9)	84.5 ± (20)	50 ± (10)	30.5 ± (6.6)	16.5 ± (2.7)	12.2 ± (2.3)	5.5 ± (0.65)
Left	T	Hind	Seq	No	1580 ± (34)	326 ± (110)	113 ± (44)	442 ± (120)	65.4 ± (21)	97.4 ± (33)	65.2 ± (19)	36.9 ± (12)	24 ± (8.2)	15 ± (4.8)	9.8± (3.2)	6.8 ± (2.7)
Left	T	Hind	TD1	No	1539 ± (63)	261 ± (52)	117 ± (11)	364 ± (63)	54.3 ± (10)	78 ± (16)	55 ± (12)	28.8 ± (6.7)	17.8 ± (3.8)	10.3 ± (2.1)	7.75 ± (1.6)	4.25 ± (0.97)
Left	L	Hind	Seq	No	1551 ± (64)	261 ± (52)	83.5 ± (25)	364 ± (63)	73 ± (3.5)	91.5 ± (5)	60 ± (17)	28.8 ± (6.7)	19.3 ± (5.1)	10.3 ± (2.1)	7.75 ± (1.6)	3.5 ± (0.87)
Left	L	Hind	Seq	yes	1500 ± (110)	306 ± (14)	60 ± (12)	310 ± (41)	73 ± (3.5)	91.5 ± (4.9)	65.5 ± (3.8)	34.5 ± (2.6)	21 ± (1.2)	12 ± (0.59)	6.5 ± (1.4)	3.5 ± (0.87)
Right	T	Front	Seq	No	1395 ± (32)	423 ±	180 ± (54)	610 ±	81.8 ± (26)	123 ± (32)	98.8 ±	58.6 ± (19)	37.9 ± (13)	22.7 ± (6.9)	14 ± (4.4)	10.2 ± (3.5)

						(110)		(120)			(33)					
Right	T	Front	TD1	No	1490 ± (190)	448 ± (180)	150 ± (21)	598 ± (150)	82.8 ± (36)	117 ± (49)	92 ± (45)	65.3 ± (32)	40.2 ± (19)	24.3 ± (9.2)	16.7 ± (5.8)	8.33 ± (2.7)
Right	L	Front	Seq	No	1570 ± (190)	450 ± (140)	135 ± (64)	558 ± (160)	97.2 ± (33)	131 ± (39)	98.8 ± (39)	46.1 ± (13)	30.5 ± (12)	19.9 ± (7.7)	12.2 ± (4.8)	7.79 ± (3.4)
Right	L	Front	Seq	yes	1570 ± (180)	348 ± (96)	97.8 ± (29)	436 ± (95)	85.8 ± (36)	110 ± (35)	72 ± (20)	39.5 ± (4.3)	24 ± (2.6)	12.8 ± (3.3)	8.5 ± (1.7)	4.83 ± (1.2)
Left	T	Front	Seq	No	1570 ± (190)	413 ± (50)	118 ± (26)	542 ± (69)	84.7 ± (10)	130 ± (16)	87 ± (9.3)	50.8 ± (17)	31.2 ± (11)	19.6 ± (5.5)	13 ± (3.9)	7.67 ± (3.4)
Left	T	Front	TD1	No	1540 ± (180)	415 ± (61)	105 ± (37)	535 ± (100)	101 ± (25)	128 ± (18)	87 ± (12)	46.7 ± (9.7)	27.3 ± (7)	16.8 ± (4.1)	12.8 ± (5.6)	6.17 ± (1.7)
Left	L	Front	Seq	No	1530 ± (130)	400 ±	85.6 ± (24)	450 ±	85.6 ± (24)	85.6 ± (24)	85.6 ±	85.6 ± (24)	85.6 ± (24)	85.6 ± (24)	85.6 ± (24)	85.6 ± (24)

						(110)		(130)			(24)					
Left	L	Front	Seq	yes	1490 ± (34)	399 ± (12)	98 ± (10)	499 ± ()	91 ± (9.3)	123.7 ± (6.1)	85.7 ± (4.4)	44 ± (1.7)	27 ± (1.2)	16.5 ± (2.7)	10.75 ± (0.97)	6 ± (1)
